# Orphan receptor GPR37L1 contributes to the sexual dimorphism of central cardiovascular control

**DOI:** 10.1186/s13293-018-0173-y

**Published:** 2018-04-06

**Authors:** James L. J. Coleman, Margaret A. Mouat, Jianxin Wu, Nikola Jancovski, Jaspreet K. Bassi, Andrea Y. Chan, David T. Humphreys, Nadine Mrad, Ze-Yan Yu, Tony Ngo, Siiri Iismaa, Cristobal G. dos Remedios, Michael P. Feneley, Andrew M. Allen, Robert M. Graham, Nicola J. Smith

**Affiliations:** 1 Molecular Pharmacology Laboratory, Division of Molecular Cardiology and Biophysics, Victor Chang Cardiac Research Institute, Darlinghurst, NSW 2010 Australia; 2 St Vincent’s Clinical School, University of New South Wales, Sydney, Australia; 3 Cardiac Biology Laboratory, Victor Chang Cardiac Research Institute, Darlinghurst, NSW Australia; 4 Cardiac Physiology & Transplantation, Victor Chang Cardiac Research Institute, Darlinghurst, NSW Australia; 5 Department of Physiology, University of Melbourne, Melbourne, Australia; 6 Florey Institute of Neuroscience and Mental Health, University of Melbourne, Melbourne, Australia; 7 Molecular, Structural and Computational Biology Division, Victor Chang Cardiac Research Institute, Darlinghurst, NSW Australia; 8 Cardiac Research Laboratory, University of Sydney, Camperdown, Australia

**Keywords:** G protein-coupled receptors, Orphan, Hypertension, Left ventricular hypertrophy, Central nervous system, Heart failure, Mouse model of hypertension

## Abstract

**Background:**

Over 100 mammalian G protein-coupled receptors are yet to be matched with endogenous ligands; these so-called orphans are prospective drug targets for the treatment of disease. GPR37L1 is one such orphan, abundant in the brain and detectable as mRNA in the heart and kidney. GPR37L1 ablation was reported to cause hypertension and left ventricular hypertrophy, and thus, we sought to further define the role of GPR37L1 in blood pressure homeostasis.

**Methods:**

We investigated the cardiovascular effects of GPR37L1 using wild-type (GPR37L1^wt/wt^) and null (GPR37L1^KO/KO^) mice established on a C57BL/6J background, both under baseline conditions and during AngII infusion. We profiled GPR37L1 tissue expression, examining the endogenous receptor by immunoblotting and a β-galactosidase reporter mouse by immunohistochemistry.

**Results:**

GPR37L1 protein was abundant in the brain but not detectable in the heart and kidney. We measured blood pressure in GPR37L1^wt/wt^ and GPR37L1^KO/KO^ mice and found that deletion of GPR37L1 causes a female-specific increase in systolic, diastolic, and mean arterial pressures. When challenged with short-term AngII infusion, only male GPR37L1^KO/KO^ mice developed exacerbated left ventricular hypertrophy and evidence of heart failure, while the female GPR37L1^KO/KO^ mice were protected from cardiac fibrosis.

**Conclusions:**

Despite its absence in the heart and kidney, GPR37L1 regulates baseline blood pressure in female mice and is crucial for cardiovascular compensatory responses in males. The expression of GPR37L1 in the brain, yet absence from peripheral cardiovascular tissues, suggests this orphan receptor is a hitherto unknown contributor to central cardiovascular control.

**Electronic supplementary material:**

The online version of this article (10.1186/s13293-018-0173-y) contains supplementary material, which is available to authorized users.

## Background

High blood pressure (BP) is responsible for > 45% of heart disease deaths worldwide and is the single biggest contributing risk factor to global disease burden [1]. The human cost of hypertension is partially explained by the disorder’s multifactorial pathogenesis and is accordingly difficult to treat [2]. Approximately 30% of patients receiving treatment for hypertension do not have their BP adequately controlled by current pharmacotherapy [3]. Clearly, a better understanding of the mechanisms governing BP is necessary to more effectively manage cardiovascular disease.

One such BP regulator may be the G protein-coupled receptor (GPCR) GPR37L1. This orphan receptor (orphan—without a known endogenous ligand) is predominantly expressed in the brain [4, 5] and contributes to cerebellar development and motor skills [6]. *Gpr37l1* also lies within eight quantitative trait loci linked to BP regulation in the rat [7]. Moreover, a previous study [8] reported that GPR37L1 knockout mice have left ventricular hypertrophy (LVH) and ~ 60 mmHg higher systolic BP than that of cardiac-specific, GPR37L1-overexpressing transgenic mice. However, the mouse strain, sex, age, heart rate, and depth of anesthesia used for BP measurement were not indicated, and BP was not compared to appropriate wild-type controls. Here, we sought to definitively establish whether GPR37L1 regulates BP using GPR37L1 null mice that we generated and maintained on a pure C57BL/6J background [9, 10]. We report that GPR37L1 is indeed involved in BP regulation but in a sexually dimorphic manner.

## Methods

### Mouse line generation and maintenance

Male and female C57BL/6J GPR37L1^wt/wt^, GPR37L1^flx/flx^, GPR37L1^wt/KO^, GPR37L1^KO/KO^, and GPR37L1^lacZ/wt^ were generated using the European Conditional Mouse Mutagenesis Program (EUCOMM) method for conditional gene inactivation using *Cre/loxP* and *Flp1/FRT* gene trapping [9] described as GOI#1 in a previous report [10]. Mice were group-housed in a temperature-controlled environment with a 12-h light-dark cycle (lights on at 0700). Standard chow and water were available ad libitum*.* All animal work was performed in accordance with the *Australia Code for the Care and Use of Animals for Scientific Purposes*, *8th Edition* (2013), and was approved by the relevant Animal Ethics Committees (Garvan Institute for Medical Research/St Vincent’s Hospital, project number AEC 13/30; University of Melbourne, project number 1212573). All animals were entered into the study in a randomized order.

### Quantitative PCR

Tissue was homogenized in TRIzol (15,596,026, Thermo Scientific, USA) using a PRO200 homogenizer (Bio-Gen, USA), and RNA was extracted using manufacturer’s protocol (Thermo Scientific). DNA contamination was removed from extracted RNA with TURBO DNAse kit treatment (AM1907, Invitrogen, USA) before cDNA synthesis was performed using SuperScript® III First-Strand Synthesis Supermix as per manufacturer’s protocol (11752050, Invitrogen). cDNA was stored at − 20 °C until use. Quantitative PCR (qPCR) was performed on cDNA using TaqMan Gene Expression Master Mix (4369016, Applied Biosystems, USA) and TaqMan probes (Applied Biosystems). All qPCR was performed using a LightCycler 480 (Roche, Switzerland) (cycling conditions: preincubation at 95 °C for 10 min; 55 amplification cycles of 95 °C for 10 s, 58 °C for 30 s, and 72 °C for 30 s; final cooling at 50 °C for 10 s). Probe Mm00661872_m1 was used to detect GPR37L1 in mouse. The following probes were used to detect components of the fetal gene program: ANP (Mm01255747_g1), BNP (Mm01255770_g1), β-MHC (Mm00600555_m1), and α-SkA (Mm00808218_g1). ΔCt values were calculated in reference to HPRT (mouse, Mm03024075_m1) or when GPR37L1 transcript abundance was determined in EUCOMM mice; ΔCt values were calculated in reference to β2M (mouse, Mm00437762_m1). Relative mRNA abundance was determined using the 2^−ΔΔCt^ method [11].

### Tissues for pilot assay of GPR37L1 transcription

In reference to the experiment shown in Additional file 1: Figure S1, surplus tissue (left and right ventricles, left and right atria, aorta, kidney, lung, liver, and brain) from sham-operated wild-type C57BL/6J mice (12 weeks old, male), as harvested for a previous study [12], was used to generate cDNA to quantify GPR37L1 by qPCR.

### Tissue fixation for β-galactosidase imaging

Tissue fixation was performed as adapted from a previous method [13]. At ≥ 12 weeks of age, GPR37L1^wt/wt^ and GPR37L1^lacZ/wt^ mice of either sex were anesthetized with 5% isofluorane in oxygen (flow rate 1 L/min) delivered via nose cone. The thoracic cavity was opened, and the LV was punctured with a needle that was then used to perfuse the heart with phosphate-buffered saline (PBS) (1 min; flow rate 20 mL/min), achieving sacrifice by exsanguination. Immediately after, tissues were fixed by further 2 min perfusion with 4% paraformaldehyde (PFA) in PBS (flow rate 20 mL/min). Following fixation, the brain, heart, and kidney were collected and further fixed in 4% PFA/PBS for 48 h at room temperature (RT). After post-fixation, tissue was stored in sucrose cryoprotectant buffer (5 mmol/L Trizma base, 77 mmol/L NaCl, 4 mmol/L Na_2_HPO_4_, 1.3 mmol/L NaH_2_PO_4_, 20% *w*/*v* sucrose) at 4 °C for ≥ 48 h. Fixed and cryoprotected tissue was embedded in Tissue Freezing Medium (TFM-5, Triangle Biomedical, USA) and frozen. Frozen tissue was cut into 12 μm sections using a CM1950 Cryostat (Leica Biosystems, Germany) and mounted on Superfrost Plus Slides (Thermofisher Scientific).

### β-galactosidase immunofluorescent staining

The protocol was performed at RT unless otherwise specified. Slides containing mounted tissue sections from the above procedure were washed for 10 min, three times, in immunobuffer [TPBS (10 mmol/L Tris, 8 mmol/L Na_2_HPO_4_, 2.6 mmol/L NaH_2_PO_4_, 0.9% NaCl, 0.05% thimerosal) with 0.3% Triton X-100]. Non-specific antibody binding was blocked by incubating each section in immunobuffer containing 10% *v*/*v* normal donkey serum (NDS) (017-000-121, Jackson ImmunoResearch Laboratories, USA) for 30 min, in a humidifying chamber. Primary antibodies [1:250 chicken anti-β-galactosidase (AB9361, Abcam, Cambridge, UK) for all tissue, 1:500 rabbit anti-GFAP (Z0334, Agilent Technologies, USA) for brain only] were diluted in immunobuffer with 10% *v*/*v* NDS and applied to each section for 16 h, in a humidifying chamber at 4 °C. Slides were then washed for 10 min, three times, in TPBS. For the remainder of the protocol, slides were shielded from ambient light. Secondary antibodies [1:500 donkey anti-chicken Cy3 (703-165-155, Jackson ImmunoResearch Laboratories) for all tissue, 1:500 donkey anti-rabbit Alexa Fluor 647 (711-605-152, Jackson ImmunoResearch) for brain only], DAPI (1:5000 4′,6-diamidino-2-phenylindole dihydrochloride; D9542, Sigma Aldrich, USA) nuclear stain, and wheat germ agglutinin (WGA) membrane stain (for heart and kidney only, 1:500 Alexa Fluor 488 WGA conjugate; W11261, Invitrogen) were diluted in immunobuffer containing 10% *v*/*v* NDS and applied to each section for 1 h, in a humidifying chamber. Slides were then washed for 10 min in TPBS. To reduce autofluorescence, each section was then treated with 0.1% *w*/*v* Sudan Black B (S2380, Sigma Aldrich) in 70% *v*/*v* ethanol for 1 h, in a humidifying chamber. Excess dye was then removed by washing for 10 min, three times, in TPBS. Slides were then dried for 16 h in a humidifying chamber before being sealed with glass coverslips mounted using 2.5% *w*/*v* DABCO (1,4-diazabicyclo-[2,2,2]-octane; D2522, Sigma Aldrich), 10% *w*/*v* polyvinyl alcohol (P8136, Sigma Aldrich), 25% *w*/*v* glycerol (G5516, Sigma Aldrich), and 100 mmol/L Tris buffer (pH 8.7), as adapted from a previous method [14]. Slides were imaged on a LSM 7 Duo confocal microscope (Zeiss, Germany).

### Immunoblotting and densitometry

Tissue homogenates and crude membranes were prepared as previously described [15, 16]. SDS-PAGE and immunoblotting was performed as previously described [15]. GPR37L1 was detected using goat anti-GPR37L1 (C-12) antibody (1:1000; sc-164532, Santa Cruz Biotechnology, USA) that was previously confirmed to be GPR37L1-specific using knockout tissue [15] and then rabbit anti-goat antibody (1:7500; 61-1620, Invitrogen). β-galactosidase was detected using rabbit anti-β-galactosidase antibody (1:5000; A-11132; Invitrogen) and then donkey anti-rabbit antibody (1:15000, NA934, GE Healthcare, Australia). GAPDH was detected using rabbit anti-GAPDH antibody (1:10000; 14C10, Cell Signaling, USA) and then donkey anti-rabbit antibody (1:20000, NA934, GE Healthcare). SuperSignal™ West Pico (34080, Invitrogen) or Clarity™ (1705060, Bio-Rad, USA) were used as chemiluminescent substrates. Densitometry was performed using ImageJ software (https://imagej.nih.gov/ij/) on non-saturated chemiluminescent exposures. Pixel density of background was measured and subtracted from subsequent measurements taken on the same image. Pixel density of GPR37L1 was measured in reference to the predominant, cleaved receptor species [15] and adjusted to equivalent protein abundance using each sample’s own GAPDH pixel density score.

### Hemodynamic measurement and analysis

At age 10–12 weeks, mice were anesthetized with 1–2% isofluorane (Zoetis, USA) in oxygen (flow rate 0.5 L/min), delivered via nose cone regulated using a mechanical ventilator (150 strokes/min, 200 μl stroke volume). Hemodynamics in the aorta and LV were recorded with a high-fidelity pressure transducer (Millar Instruments, USA) following its insertion into the right carotid artery as described previously [17, 18]. Heart rate was maintained at approximately 500 beats per minute (bpm) by manual adjustment of the isofluorane concentration. Data were recorded and analyzed using AcqKnowledge software (version 3.9.0 for Windows, BIOPAC Systems, USA) by an observer blinded to genotype (and to treatment where applicable). Hemodynamics on osmotic mini pump animals were recorded using a Transonic Scisense (FTH-1211B-001, USA) high-fidelity pressure transducer.

### Radiotelemetry blood pressure measurement

At 12–14 weeks of age, mice were anesthetized with 2% isofluorane (Abbott Laboratories, USA), with the rate adjusted to provide a deep surgical plane. PA-C10 telemeters (Data Sciences International, USA) were implanted as previously described [19], with the pressure-sensing catheter inserted into the left carotid artery. Analgesia was administered before and after surgery (carprofen, 0.5 mg/100 g, IP; Norbrook, UK). Hydration was maintained by administration of 0.5 mL 0.9% NaCl (IP), given at the time of operation and again 24 h later. Approximately 2 weeks after telemeter implantation, blood pressure monitoring was performed twice daily in the home cage, between 09:00 and 12:00 (light phase) and between 21:00 and 24:00 (dark phase) for 9 days. The average of the 9 days of measurements in each phase was taken to give a single value per animal per phase. [N.B. radiotelemetry blood pressure monitoring was only performed during initial phenotyping experiments in Fig. 2c, d, as indicated].

### Angiotensin II infusion

Osmotic mini pumps (product 1007D, Alzet) were filled with either vehicle (0.15 mol/L NaCl, 1 mmol/L acetic acid) or AngII (A9525, Sigma Aldrich) at a concentration adjusted to allow 2 mg/kg/day release of AngII for 7 days. After filling, pumps were primed by incubation in 0.9% NaCl for 16 h at 37 °C. Mice (10–12 weeks old) were anesthetized [inhaled 3–4% isofluorane (Zoetis) in oxygen (flow rate 0.8–1.0 L/min) delivered by nose cone] and pumps implanted subcutaneously, as per the manufacturer's instructions. Following pump implantation, topical analgesia was administered in the form of bupivacaine (AstraZeneca, UK), and systemic analgesia was administered (SC) in the form of buprenorphine (0.075 mg/kg; Reckitt Benckiser, UK), before completion of surgery and recovery from anesthesia. Animals were allowed to recover from anesthesia on a heated pad, before being single-housed for the remainder of the study. [N.B. blood pressure following the 7 days of infusion was measured by anesthetized catheterization, as indicated].

### Morphometry

Tissues were retrieved after sacrifice, briefly rinsed of blood in 0.9% NaCl, and then blotted dry. Tissue weights were measured immediately and expressed as a ratio of each animal’s own tibia length, measured with caliMAX fine calipers (WIHA, Germany).

### Cardiomyocyte density

Cardiomyocyte density was determined using an adaptation from a previous method [18]. Briefly, LV tissue was fixed in 4% PFA/PBS for 24 h at RT before post-fixation, sectioning, and processing as described above for immunofluorescent staining and confocal microscopy. Cell membranes were visualized using 1:500 Alexa Fluor 488 WGA conjugate. Using ImageJ, an observer blinded to sex, genotype, and treatment counted cardiomyocytes from two representative areas of each LV and averaged them to give one value per animal. Cardiomyocyte counts were expressed as cells per square millimeter. Cardiomyocyte density was used instead of cardiomyocyte cross-sectional area as we found density to show less inter-observer variability (data not shown).

### Fibrosis staining

LV tissue was fixed as described for cardiomyocyte density. Cytoplasm (red) and collagen (blue) were stained using Masson’s trichrome stain as per the manufacturer’s protocol (HT15, Sigma Aldrich). Coverslips were mounted onto slides using DEPEX mounting media (VWR International, USA). Sections were visualized with bright-field microscopy on a DM6000 power mosaic microscope (Leica Biosystems), and images were retained in RGB format. The degree of fibrosis was quantified using ImageJ by an observer blinded to sex, genotype, and treatment and expressed as a percentage of area occupied by blue-hued pixels relative to total cross-sectional tissue area, as adapted from previous methods [20].

### RNA sequencing analysis

Human postmortem brain RNA sequencing data was obtained from the Genotype-Tissue Expression (GTEx) Consortium [21]. GTEx raw sequence counts were examined for differentially expressed genes using the edgeR statistical package [22], where samples were grouped by genders for each brain tissue sample.

### Statistical analysis

All data are presented as mean ± s.e.m. The number of independent observations (*n*) in animal/human data represents a single animal/donor. Where multiple measurements were taken per animal, measurements were averaged to give a single observation (*n*) per animal. Where two untreated genotypes (per sex) were analyzed, a two-tailed Student’s *t* test was used, or a one-tailed Mann-Whitney test, as indicated. Where > 2 untreated genotypes (per sex) were analyzed, a one-way ANOVA was used with Dunnett’s multiple comparisons tests, or Kruskal-Wallis test with Dunn’s multiple comparisons test where ANOVA assumptions were violated. Where two genotypes (per sex), with and without treatment, were analyzed, a two-way ANOVA was used with Tukey’s multiple comparisons test. For all tests, *p* ≤ 0.05 was considered significant.

## Results

### GPR37L1 protein is abundant in the central nervous system but not detectable in the heart or kidney

**Fig. 1:**
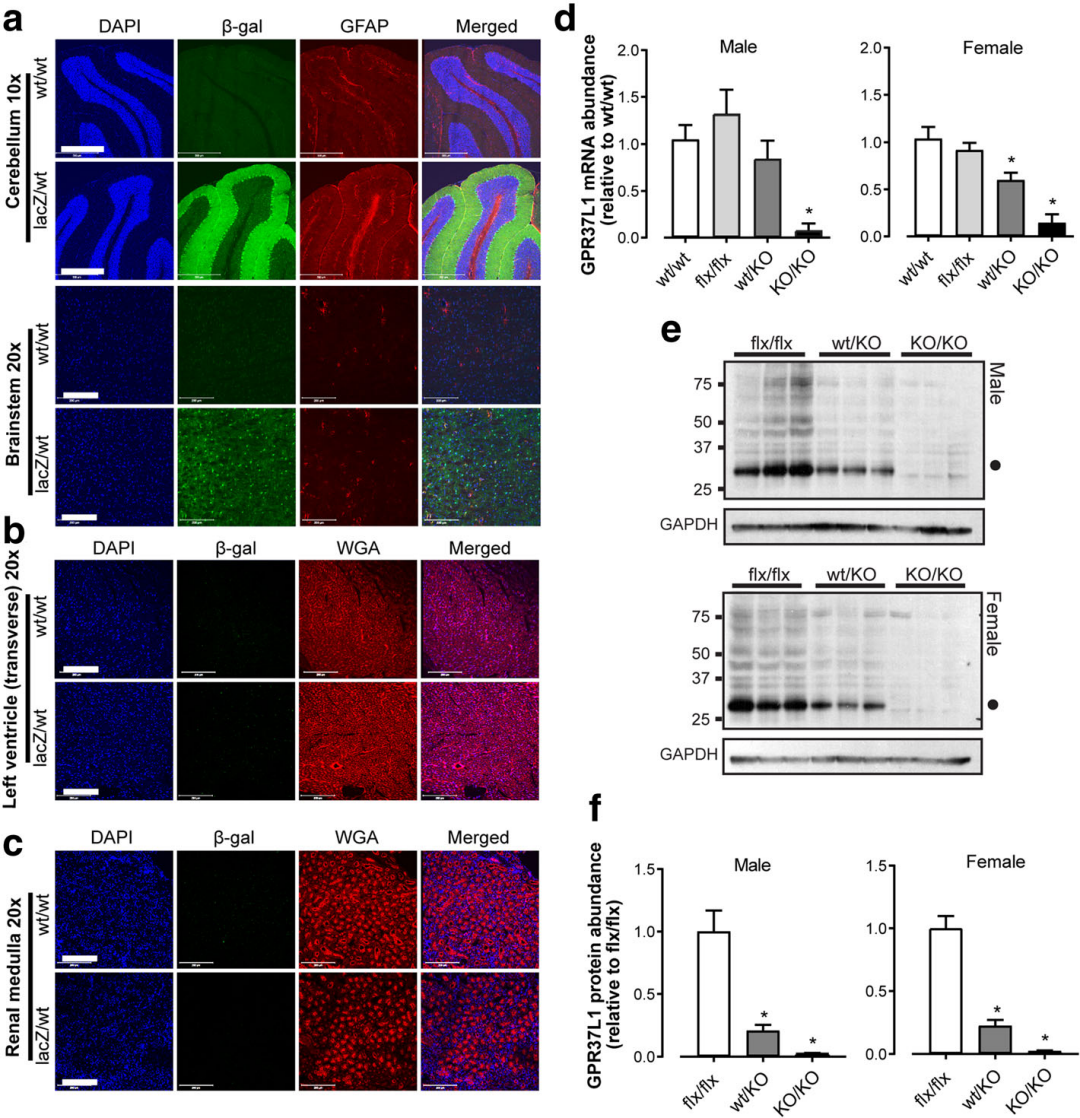
GPR37L1 is abundant in the brain and ablated in EUCOMM-derived knockout mice. **a**–**c** Paraformaldehyde-fixed tissue from GPR37L1^lacZ/wt^ and GPR37L1^wt/wt^ mice (mixed-sex, ≥ 12 weeks old) **a** Brain: nuclei (blue; DAPI); GPR37L1 β-galactosidase reporter (green; β-galactosidase antibody); astrocytes and the Bergmann glia [red, glial fibrillary acidic protein antibody (GFAP)]. Scale bars: 500 μm (× 10 images) and 10 μm (× 20 images). **b** Heart and **c** kidney: nuclei (blue, DAPI); GPR37L1 β-galactosidase reporter (green; β-galactosidase antibody); cell membranes [red; wheat germ agglutinin (WGA)]. Images representative of *n* = 3. Scale bar = 200 μm. **d** qPCR confirmed loss of cerebellar GPR37L1 mRNA in male and female GPR37L1^KO/KO^ mice (10–12 weeks old, *n* = 6, one-way ANOVA with Dunnett’s multiple comparisons, *p ≤ 0.05). **e** Immunoblot and **f** densitometry of GPR37L1 in cerebellar homogenates from male and female mice (genotypes as indicated, 10–12 weeks old, *n* = 3, one-way ANOVA with Dunnett’s multiple comparisons, *p ≤ 0.05). Closed circle (*M*_r_ ~ 30 kD) indicates predominant, cleaved GPR37L1 species [15]

Given the reported role of GPR37L1 in the cardiovascular system [8], we investigated GPR37L1 transcription by qPCR in several mouse tissues of interest, including the heart and kidney. We noted that GPR37L1 mRNA appeared at late cycle numbers in both tissues, suggesting very low relative expression (Additional file 1: Figure S1). To clarify the expression profile of GPR37L1, we used EUCOMM ES cells [9] to generate GPR37L1^lacZ/wt^ mice (expressing one allele of β-galactosidase in place of GPR37L1) as we have described [10]. Consistent with previous reports of GPR37L1 localization [4–6, 23], β-galactosidase expression was most abundant in the brain, particularly in the cerebellar Bergmann glia (Fig. 1a, Additional file 1: Figure S2A). Astrocyte β-galactosidase co-localization was observed throughout the brain but was most evident in the hippocampus (Additional file 1: Figure S2B), while other regions such as the brain stem (Fig. 1a) and neocortex (Additional file 1: Figure S2C) contained GFAP-negative β-galactosidase-positive cells that were consistent with the reported expression of GPR37L1 in cells of oligodendrocyte lineage [23, 24]. In several key cardiovascular centers of the CNS including the caudal and rostral ventrolateral medulla (CVLM, RVLM), the nucleus of the solitary tract (NTS), and the A5 nucleus, diffuse β-galactosidase staining was observed close to, but not within, tyrosine hydroxylase-positive catecholaminergic neurons (Additional file 1: Figure S3). In contrast, we were unable to detect GPR37L1 β-galactosidase reporter immunofluorescence in peripheral tissues. Although we measured low levels of GPR37L1 mRNA in the mouse heart (Additional file 1: Figure S1), we could not detect β-galactosidase by immunofluorescence (Fig. 1b, Additional file 1: Figure S4A) or Western blot (Additional file 1: Figure S4B), nor wild-type GPR37L1 using a validated [15] anti-GPR37L1 antibody (Additional file 1: Figure S4C-E). Similarly, we did not detect β-galactosidase in the renal medulla (Fig. 1c) or cortex (Additional file 1: Figure S5A), nor could it be detected in GPR37L1^lacZ/wt^ kidney homogenates (Additional file 1: Figure S5B). Endogenous GPR37L1 was not detected in GPR37L1^wt/wt^ kidney (Additional file 1: Figure S5C). Because only GPR37L1 mRNA, but not protein, was found in the heart and kidney, we hypothesize that GPR37L1 exerts its cardiovascular effect via the CNS.

### GPR37L1 regulates blood pressure in female mice

To investigate the role of GPR37L1 in the cardiovascular system, we further generated the GPR37L1^flx/flx^ and GPR37L1^KO/KO^ mice [9, 10]. Receptor ablation in both genders was verified in the cerebellum (mRNA and protein, respectively; Fig. 1d–f), and in the LV (mRNA; Additional file 1: Figure S4F). GPR37L1 expression was unchanged in GPR37L1^flx/flx^ as compared to GPR37L1^wt/wt^ mice (generated from GPR37L1^wt/KO^ breeding pairs) (Fig. 1d, Additional file 1: Figure S6A and B). Thus, GPR37L1^wt/wt^ mice were used as controls for the remainder of our studies.

**Fig. 2:**
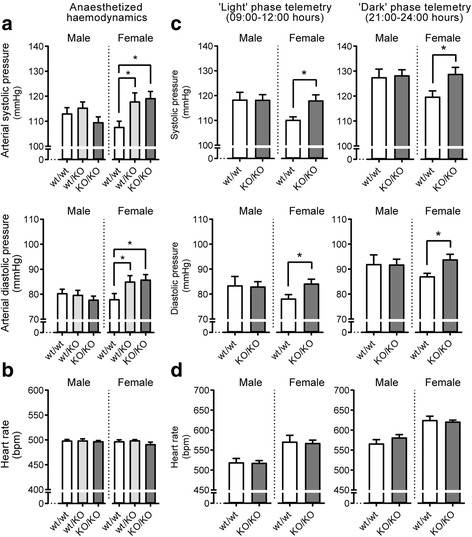
Female GPR37L1 knockout mice have elevated blood pressure. **a** Micromanometry under anesthesia was performed on male and female mice (10–12 weeks old, *n* ≥ 13, one-way ANOVA with Dunnett’s post hoc test, or Kruskal-Wallis with Dunn’s multiple comparisons where ANOVA assumptions were violated. *p ≤ 0.05). **b** Heart rate did not differ between genotypes during recordings. **c** Conscious radiotelemetry in male and female mice. Data represents averaged recordings during light and dark phases of diurnal cycle over 9 days (14–16 weeks old, *n* = 5, *p ≤ 0.05, two-tailed Student’s *t* test between genotypes within each sex). **d** Heart rate did not differ between genotypes during conscious recordings

Baseline cardiovascular measures were first assessed in anesthetized females by micromanometry (Fig. 2a) at a constant heart rate of ~ 500 bpm (Fig. 2b, Additional file 1: Table S1). Female GPR37L1^wt/KO^ and GPR37L1^KO/KO^ mice displayed significantly elevated aortic systolic pressure compared to controls (Fig. 2a, Additional file 1: Table S1). Furthermore, GPR37L1^KO/KO^ females showed increased aortic diastolic, mean arterial, and pulse pressures (Fig. 2a, Additional file 1: Table S1). No genotype differences in LV systolic pressure, end-diastolic pressure, and dP/dT max or dP/dT min were observed. To confirm that this phenotype was preserved in conscious animals, radiotelemetry BP monitoring was performed on a separate cohort of conscious GPR37L1^wt/wt^ and GPR37L1^KO/KO^ females. Again, GPR37L1^KO/KO^ females had elevated systolic and diastolic pressure, during both light and dark phases of the diurnal cycle, relative to GPR37L1^wt/wt^ controls (Fig. 2c).

**Fig. 3:**
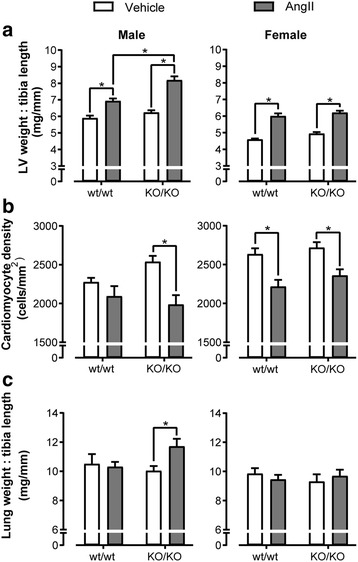
Male GPR37L1^KO/KO^ mice have an exacerbated response to AngII infusion. Effect of 7 days AngII infusion (2 mg/kg/day) on male and female GPR37L1^wt/wt^ and GPR37L1^KO/KO^ mice (10–12 weeks old). **a** Left ventricle (LV) weight normalized to tibia length. *n* ≥ 15. **b** Cardiomyocyte hypertrophy calculated as cardiomyocytes per square millimeter. Representative images in Additional file 1: Figure S7. *n* ≥ 6. **c** Lung weight normalized to each animal’s own tibia length. For all panels, two-way ANOVA results are displayed in Additional file 1: Table S3, and *p ≤ 0.05 according to Tukey’s multiple comparisons test

**Fig. 4:**
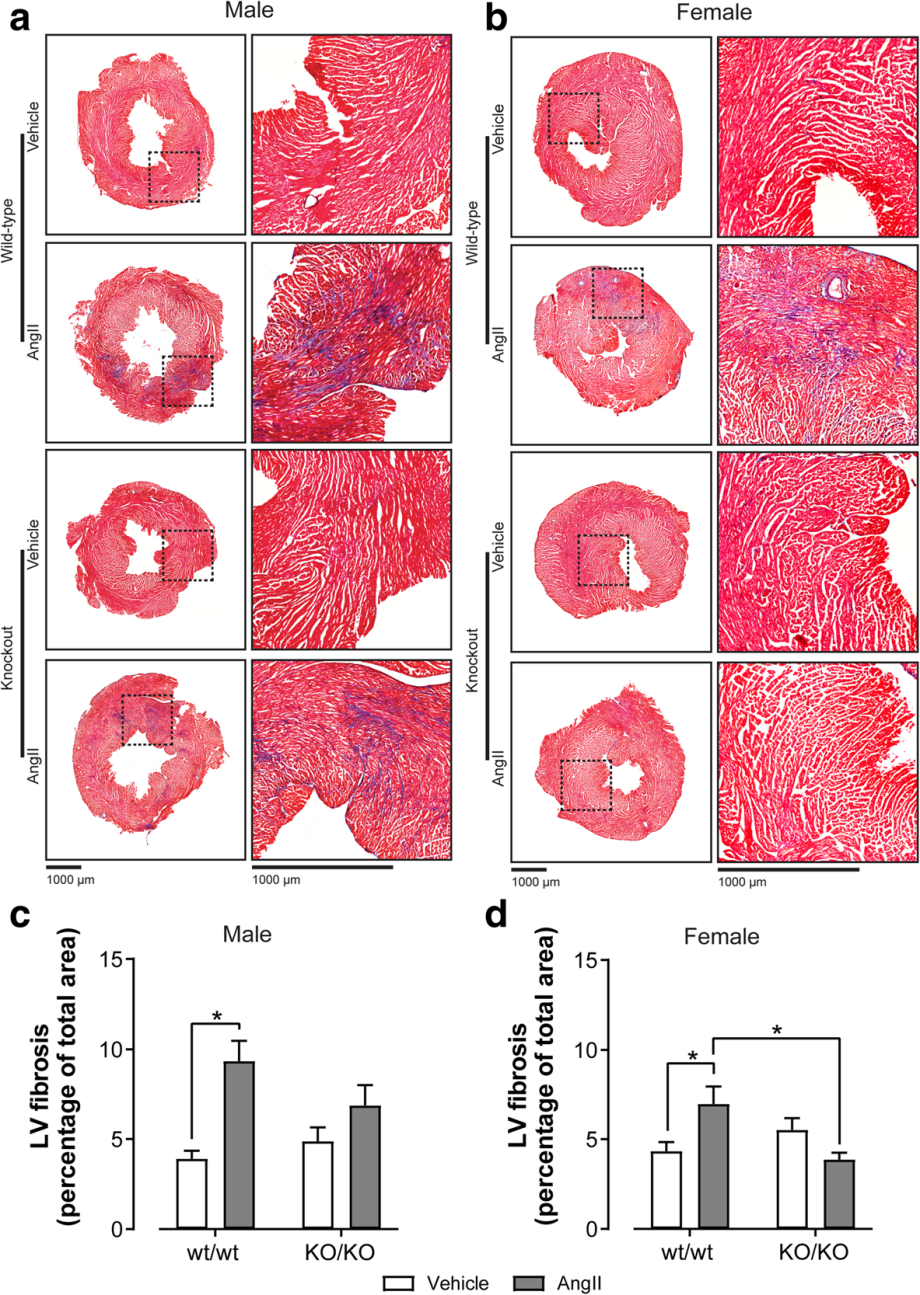
GPR37L1^KO/KO^ mice are protected from AngII-induced cardiac fibrosis. Images of paraformaldehyde-fixed LV tissue from male (**a**) and female (**b**), vehicle- and AngII-treated GPR37L1^wt/wt^ and GPR37L1^KO/KO^ mice were cut into 10-μm sections and stained with Masson’s trichrome to highlight fibrosis (blue) and total tissue (red). Representative images of *n* ≥ 6. Scale bar size as indicated on figure. Cardiac fibrosis was quantified and expressed as a percent area of total cross-sectional LV area for males (**c**) and females (**d**). *n* ≥ 6, two-way ANOVA results displayed in Additional file 1: Table S3, and *p ≤ 0.05 according to Tukey’s multiple comparisons test

To investigate if GPR37L1 deletion is deleterious in the setting of hypertensive stress, female GPR37L1^wt/wt^ and GPR37L1^KO/KO^ mice were infused for 7 days with AngII (2 mg/kg/day) or vehicle, before anesthetized hemodynamic measurement by pressure-transducing catheter and tissue collection. AngII infusion caused greater increases in systolic and mean arterial pressure in the GPR37L1^wt/wt^ females (Table 1), presumably because GPR37L1^KO/KO^ females have higher baseline systolic pressure (one-tailed Mann-Whitney test, *p* ≤ 0.05), consistent with our previous, independent cohort (Fig. 2). However, BP after 7 days of AngII did not differ between genotypes (Table 1), and both developed equivalent gross, cellular, and molecular LVH (Fig. 3a, b, Additional file 1: Figure S7, Figure S8), without progression to overt heart failure (see relative lung and right ventricle weights; Fig. 3c, Table 1). Although GPR37L1^wt/wt^ females developed cardiac fibrosis in response to AngII treatment, this was not observed in GPR37L1^KO/KO^ females (Fig. 4).

**Table 1:** Hemodynamic assessment of AngII-treated (2 mg/kg/day) female GPR37L1^wt/wt^ and GPR37L1^KO/KO^ mice by micromanometry under anesthesia

		Female					
	Genotype	wt/wt			KO/KO		
	Treatment	Vehicle	AngII	Δ	Vehicle	AngII	Δ
Aorta	HR (bpm)	515.6 ± 8.0	512.3 ± 7.1	− 3.3	504.4 ± 6.8	503.6 ± 3.6	− 0.8
	SP (mmHg)	109.3 ± 3.3	135.5 ± 4.1*	26.2	119.7 ± 6.3	130.9 ± 3.7	11.2
	DP (mmHg)	78.1 ± 2.3	83.7 ± 2.5	5.6	83.1 ± 2.8	86.8 ± 2.4	3.7
	MAP (mmHg)	88.5 ± 2.6	101 ± 2.8*	12.5	95.3 ± 3.9	101.5 ± 2.7	6.2
	PP (mmHg)	31.2 ± 1.2	51.8 ± 3.0*	20.6	36.6 ± 3.6	44.0 ± 2.2	7.4
LV	HR (bpm)	500.9 ± 8.2	500.4 ± 2.2	− 0.5	514.0 ± 9.6	508.9 ± 4.8	− 5.1
	SP (mmHg)	106.5 ± 3.1	128.8 ± 1.5*	22.3	112.9 ± 4.7	126.4 ± 2.6*	13.5
	EDP (mmHg)	6.4 ± 0.5	7.0 ± 1.1	0.6	5.3 ± 0.3	8.7 ± 1.1*	3.4
	dP/dT max (mmHg/s)	9799 ± 860.0	10,998 ± 426.2	1199	11,343 ± 745	12,455 ± 383.9	1112
	dP/dT min (mmHg/s)	− 9019 ± 853.7	− 9253 ± 584.0	− 234	− 9262 ± 470.2	− 9718 ± 330.8	− 456
Morphometry	Body weight (g)	21.2 ± 0.3	22.3 ± 0.3	1.1	23.3 ± 0.5	22.3 ± 0.3	−1.0
	Tibia length (mm)	16.1 ± 0.1	16.1 ± 0.1	0	16.4 ± 0.1	16.4 ± 0.1	0
	HW:TL (mg/mm)	6.5 ± 0.1	8.1 ± 0.2*	1.6	6.8 ± 0.1	8.2 ± 0.2*	1.4
	LVW:TL (mg/mm)	4.6 ± 0.1	6.0 ± 0.2*	1.4	4.9 ± 0.1	6.2 ± 0.1*	1.3
	RVW:TL (mg/mm)	1.4 ± 0.0	1.4 ± 0.0	0	1.4 ± 0.0	1.4 ± 0.0	0
	Lung:TL (mg/mm)	9.9 ± 0.4	9.5 ± 0.3	− 0.4	9.3 ± 0.5	9.7 ± 0.4	0.4

The two-way ANOVA results are displayed in Additional file 1: Table S3. The italics and the asterisk (*) indicate a significant (*p* ≤ 0.05) difference between AngII and vehicle-treated cohorts, within each genotype (Tukey’s multiple comparisons test). The italics and the dagger (†) indicate a significant (*p* ≤ 0.05) difference between AngII-treated animals of different genotypes (Tukey’s multiple comparisons test). Hemodynamic data, *n* ≥ 8; morphometry data, *n* ≥ 18

*HR* heart rate, *SP* systolic pressure, *DP* diastolic pressure, *MAP* mean arterial pressure, *PP* pulse pressure, *ED* end-diastolic pressure, *HW* heart weight, *LVW* left ventricle weight, *TL* tibia length, *LV* left ventricle, *RVW* right ventricle weight

### GPR37L1 protects against AngII cardiovascular stress in male mice

Baseline cardiovascular measures were also assessed in males. GPR37L1^wt/wt^, GPR37L1^wt/KO^ and GPR37L1^KO/KO^ males showed no differences in any hemodynamic parameter by micromanometry (Fig. 2a, Additional file 1: Table S1). BP radiotelemetry was performed on a separate cohort of conscious male GPR37L1^wt/wt^ and GPR37L1^KO/KO^ animals. Consistent with previous reports [25], males had higher systolic and diastolic BP than females (Fig. 2c) and lower heart rates (Fig. 2d). The expected circadian dip was seen in both sexes (Fig. 2c).

In contrast to the females, hypertension was not sustained in male mice after 7 days of AngII infusion (Table 2), as measured by pressure-transducing catheter under anesthesia, although it was clear from the various physiological endpoints that the mice received pressure overload. While AngII treatment induced LVH for males of both genotypes, the extent of this enlargement was significantly greater in GPR37L1^KO/KO^ mice (Fig. 3a, Table 2). Cardiomyocyte density reflected this difference, with only GPR37L1^KO/KO^ LV sections indicating cardiomyocyte hypertrophy in response to AngII (Fig. 3b, Additional file 1: Figure S7). In concert with exacerbated LVH, male AngII-treated GPR37L1^KO/KO^ mice had increased relative lung weight (Fig. 3c), indicative of pulmonary edema resulting from congestive heart failure [26–28] and suggesting that GPR37L1^KO/KO^ males are susceptible to AngII-induced heart failure. Indeed, we observed a blunted contractile response in AngII-treated GPR37L1^KO/KO^ males compared to GPR37L1^wt/wt^ counterparts (Table 2). Consistent with our previous cohort (Fig. 2), the baseline aortic systolic pressure of male GPR37L1^KO/KO^ did not differ from that of GPR37L1^wt/wt^ controls (one-tailed Mann-Whitney test, *p* > 0.05) (Table 2).

**Table 2:** Hemodynamic assessment of AngII-treated (2 mg/kg/day) male GPR37L1^wt/wt^ and GPR37L1^KO/KO^ mice by micromanometry under anesthesia

		Male					
	Genotype	wt/wt			KO/KO		
	Treatment	Vehicle	AngII	Δ	Vehicle	AngII	Δ
Aorta	HR (bpm)	487.2 ± 11	498.5 ± 13.3	11.3	486. ± 7.2	506.3 ± 19.4	20.3
	SP (mmHg)	114 ± 2.8	127.1 ± 6.5	13.1	107.9 ± 4.4	118.5 ± 6.3	10.6
	DP (mmHg)	78.4 ± 1.3	82.8 ± 4.6	4.4	75.4 ± 3.4	77.3 ± 6.0	1.9
	MAP (mmHg)	90.3 ± 1.6	97.6 ± 5.2	7.3	86.3 ± 3.6	91.0 ± 6.0	4.7
	PP (mmHg)	35.6 ± 2.2	44.3 ± 2.7	8.7	32.5 ± 2.3	41.2 ± 2.6	8.7
LV	HR (bpm)	490.5 ± 21.7	513.6 ± 9.4	23.1	493.5 ± 8.1	493.5 ± 12.1	0
	SP (mmHg)	113.8 ± 4.0	127.6 ± 6.3	13.8	109.5 ± 2.2	114.3 ± 5.2	4.8
	EDP (mmHg)	7.6 ± 1.0	7.3 ± 0.5	− 0.3	5.8 ± 0.5	9.9 ± 3.0	4.1
	dP/dT max (mmHg/s)	10,123 ± 953.3	12,844 ± 605.2	2721	8772 ± 438.1	*9413 ± 733.1*†	641
	dP/dT min (mmHg/s)	− 9753 ± 1024	− 8684 ± 650.6	1069	− 8517 ± 736.7	− 7653 ± 594.6	864
Morphometry	Body weight (g)	28.5 ± 0.5	25.7 ± 0.4*	− 2.8	29.4 ± 0.5	26.5 ± 0.3*	− 2.9
	Tibia length (mm)	16.6 ± 0.1	16.6 ± 0.1	0	16.7 ± 0.1	16.7 ± 0.1	0
	HW:TL (mg/mm)	8.3 ± 0.2	9.3 ± 0.2*	1.0	8.6 ± 0.2	10.9 ± 0.4*†	2.3
	LVW:TL (mg/mm)	5.9 ± 0.2	6.9 ± 0.5*	1.0	6.2 ± 0.1	8.2 ± 0.2*†	2.0
	RVW:TL (mg/mm)	1.7 ± 0.1	1.6 ± 0.1	− 0.1	1.8 ± 0.0	1.7 ± 0.1	− 0.1
	Lung:TL (mg/mm)	10.5 ± 0.7	10.3 ± 0.3	− 0.2	10.0 ± 0.3	11.7 ± 0.5*	1.7

The two-way ANOVA results are displayed in Additional file 1: Table S3. The italics and the asterisk (*) indicate a significant (*p* ≤ 0.05) difference between AngII and vehicle-treated cohorts, within each genotype (Tukey’s multiple comparisons test). The italics and the dagger (†) indicates a significant (*p* ≤ 0.05) difference between AngII-treated animals of different genotypes (Tukey’s multiple comparisons test). Hemodynamic data, *n* ≥ 7; morphometry data, *n* ≥ 15

*HR* heart rate, *SP* systolic pressure, *DP* diastolic pressure, *MAP* mean arterial pressure, *PP* pulse pressure, *EDP* end-diastolic pressure, *HW* heart weight, *LVW* left ventricle weight, *TL* tibia length, *LV* left ventricle, *RVW* right ventricle weight

As for the females, GPR37L1^KO/KO^ males appeared to be protected from myocardial fibrosis (Fig. 4a, c). However, the more marked hypertrophy and therefore increased tissue area in the GPR37L1^KO/KO^ males may have obscured the extent of absolute fibrosis which was not grossly different between genotypes (Fig. 4a).

## Discussion

Here, we evaluated the cardiovascular effects of GPR37L1 using a gene inactivation mouse model. We found that BP was increased only in female GPR37L1^KO/KO^ mice, compared to GPR37L1^wt/wt^ counterparts. In contrast, AngII-induced LVH was exacerbated only in male GPR37L1^KO/KO^ mice and was associated with depressed contractility (reduced dP/dT max) and heart failure (increased relative lung weight). Using a range of approaches, we have observed that the orphan receptor GPR37L1 is predominantly expressed in glial cells of the brain, yet we could not detect the protein in peripheral tissues. Taken together, these findings indicate GPR37L1 is an important central regulator of BP homeostasis but that its cardiovascular effects differ in males and females.

We show that female GPR37L1^KO/KO^ mice had ~ 12 mmHg greater systolic BP than GPR37L1^wt/wt^ counterparts. Such an increase in BP is not trivial, as it would translate to a doubling in cardiovascular disease risk if it were observed in humans [29]. Basal BP was unaltered in GPR37L1^KO/KO^ males, but they failed to mount compensatory responses to AngII-induced hypertensive stress, exhibiting heightened LVH, impaired LV contractile function, and lung congestion. The most parsimonious interpretation of our findings is that GPR37L1^KO/KO^ males had an exaggerated pressor response to AngII. However, we did not observe sustained AngII hypertension in males of either genotype, despite each having obvious LVH, and this is a potential limitation of the present study. Yet, given that LVH is known to increase in proportion to BP in both mouse and human hypertension [30, 31], and that males are more susceptible to AngII-induced hypertension than females [32], the data suggest that AngII induced pressure overload in males of both genotypes, causing systolic dysfunction, as seen in previous rodent pressure overload studies [33]. Indeed, only males lost body weight in response to AngII, supportive of AngII being more detrimental to their health (Table 2). This broadly agrees with human heart failure epidemiology—low systolic pressure and LV function at hospital admission is more common in men [34]. Further, systolic function is better preserved in women with aortic stenosis than men, despite equivalent hypertensive stress [35]. Interestingly, GPR37L1^KO/KO^ females appeared to be protected from cardiac fibrosis in response to AngII, despite their equivalent LVH and BP following infusion. The lack of detectable GPR37L1 protein in the heart suggests that, unlike the direct, pro-fibrotic effects of cardiac-expressed GPCRs (e.g., the β_2_ adrenoceptor, AT_1_ and ET_A_ receptors [36]), GPR37L1 may indirectly influence fibrosis by central modulation of circulatory factors that regulate the extracellular matrix, though this remains to be investigated. Alternatively, it is possible that the reduced cardiac fibrosis is indirectly linked to LV GPR37L1 transcript, rather than to protein, through regulation of miRNA, which are known to play roles in cardiac fibrosis [37]. Ultimately, detailed studies using radiotelemetry in concert with echocardiography are required to fully evaluate temporal changes in hemodynamic response to AngII, and the mechanism of decompensation in the GPR37L1^KO/KO^ male mice, and whether GPR37L1 conveys cardioprotection in other cardiovascular disease models (i.e., thoracic aortic constriction, myocardial infarction). Such studies are currently underway.

Our observation of sexual dimorphism in the cardiovascular effects of GPR37L1 is intriguing, although sex differences in the cardiovascular control systems are known to contribute to differential disease etiology and outcomes [38, 39]. While GPR37L1 had previously been linked to hypertension [8], the animals’ sex was not reported. Meanwhile, previous studies of GPR37L1 in the brain investigated only male mice [6, 40] or did not disclose sex [23]. Notably, sexual dimorphism has been reported for GPR37, the closest phylogenetic neighbor of GPR37L1 [41], and shown to be more abundant in human females in a variety of brain sub-regions at different developmental stages [42]. Further, GPR37 deletion in mice leads to sex-dependent effects on neurotransmitter levels and anxiety [43]. In our study, we did not see significant gender differences in cerebellar GPR37L1 mRNA or protein levels (Additional file 1: Figure S9A–C). Additionally, our analysis of human postmortem brain RNA-sequencing (collected by the Genotype-Tissue Expression Consortium [21]) shows the cortex to be the only sub-region with sex differences in GPR37L1 mRNA abundance, which is increased in females (Additional file 1: Figure S9D, Table S4). Taken together, this suggests that GPR37L1 may exert its gender-specific effects on BP via a mechanism that is independent of its expression level. Such effects might involve hormonal influences; estrogen, for example, is well known to sensitize central baroreflex and modulate sympathetic nerve activity [44].

Sex differences alone cannot reconcile the BP phenotype of GPR37L1 deletion in the present study (+ 12 mmHg, female-specific) with that previously reported (+ 60 mmHg, sex not disclosed) [8]. Although both studies determined BP in anesthetized animals, our additional baseline BP measures by radiotelemetry in conscious, free-moving mice show that the ~ 12 mmHg increase in GPR37L1^KO/KO^ females is independent of heart rate and anesthesia response. The magnitude of the previously reported GPR37L1 hypertensive phenotype [8] is also difficult to reconcile with the effects of deletion of other major cardiovascular regulators. For example, deletion of regulator of G protein signaling 2 [45], endothelial nitric oxide synthase [46], adenosine A_2A_ receptor [47], and bradykinin B2 receptor [48] reportedly increased BP by 45, 28, 25, and 20 mmHg, respectively. Therefore, it is likely that the effect of GPR37L1 on BP was overestimated due to the lack of comparisons to wild-type controls. Moreover, we obtained the same reported GPR37L1 knockout mouse line [8] (reanimated from frozen sperm by Charles River Laboratories, Japan) and failed to detect a change in BP by tail cuff plethysmography in a small cohort, suggesting that any BP difference in these mice is also likely to be modest (data not shown). We additionally found these mice to be of mixed genetic background. Such confounding genetic issues were avoided in our study by the use of EUCOMM conditional-ready mice [9, 10], enabling comparison of wild-type and null mice on the same C57BL/6J background.

How then might GPR37L1 regulate cardiovascular function? While our laboratory has shown GPR37L1 to constitutively couple to the Gα_s_ G protein [15], and to share antagonists with orexin and bombesin GPCRs [49], the lack of a high-affinity ligand means pharmacological probing of its physiology is intractable at present. GPR37L1 expression in the brain is well established, while its role and presence in the periphery remains unclear [41]. For example, absence of GPR37L1 mRNA in human heart and kidney has been reported [4], while another study reported low levels of GPR37L1 mRNA in both tissues in the rat [50]. Here, we also detected GPR37L1 mRNA at very low levels in the heart and kidney but could not detect GPR37L1 protein in either. Our data agree with that of the Human Protein Atlas [51], which reports no expression of GPR37L1 protein outside the brain. While vascular function remains to be explored in GPR37L1^KO/KO^ mice, we did not observe any vascular β-galactosidase immunoreactivity in the heart and kidney of GPR37L1^lacZ/wt^ mice, and the RNA abundance of GPR37L1 in the human artery and aorta is comparable to that of the heart and kidney [21], where we could not detect receptor protein. This raises the fascinating possibility that GPR37L1 is a previously undiscovered component of the CNS-cardiovascular axis. Widely expressed in astrocytes throughout the brain/brainstem, GPR37L1 may exert cardiovascular control through its proximity to autonomic control regions such as the CVLM, RVLM, and NTS [52], where astrocytes contribute to the brain renin-angiotensin system [53, 54]. Alternatively, GPR37L1 could be involved in cerebellar circuits known to influence the cardiovascular system [55, 56]. Indeed, the cerebellum has a long-suspected role in the modulation of baroreflexes and the development of essential hypertension [57, 58]. More speculatively, cerebellar control of respiration [59] may exert secondary influences on BP, with a recent study implicating excessive respiratory modulation by brainstem neurons in the development of rat and human hypertension [60].

## Conclusions

The present study found that deletion of the orphan GPCR, GPR37L1, causes increased systolic and diastolic blood pressure in female mice. In males, however, deletion of GPR37L1 blunted a compensatory response to AngII challenge. Although we observed low levels of GPR37L1 mRNA in the mouse heart and kidney, we could not detect GPR37L1 protein in either organ. Thus, while the mechanism by which GPR37L1 regulates BP in a sexually dimorphic manner has yet to be determined, it is likely its role in cardiovascular homeostasis is confined to its CNS actions, where the protein is abundant. Thus, the present study suggests that GPR37L1 is a contributor to CNS cardiovascular control, and the sex differences therein, and may contribute to the role of the CNS in hypertension and heart failure.

## Additional file

Additional file 1: List of Supplemental Digital Content. (PDF 2673 kb)

## Abbreviations

AngII: Angiotensin II
ANP: Atrial natriuretic peptide
BNP: Brain natriuretic peptide
BP: Blood pressure
CNS: Central nervous system
CVLM: Caudal ventrolateral medulla
DAPI: 4′,6-Diamidino-2-phenylindole dihydrochloride
EUCOMM: European Conditional Mouse Mutagenesis Program
GAPDH: Glyceraldehyde 3-phosphate dehydrogenase
GFAP: Glial fibrillary acidic protein
GPCR: G protein-coupled receptor
GTEx: Genotype-Tissue Expression Consortium
HPRT: Hypoxanthine guanine phosphoribosyl transferase
LV: Left ventricle
LVH: Left ventricular hypertrophy
NDS: Normal donkey serum
NTS: Nucleus of the solitary tract
PFA: Paraformaldehyde
qPCR: Quantitative polymerase chain reaction
RT: Room temperature
RVLM: Rostral ventrolateral medulla
SC: Subcutaneous
WGA: Wheat germ agglutinin
α-SkA: α-skeletal actin
β2M: β-2 microglobulin
β-MHC: β-myosin heavy chain
