# Supplementary material for: Orphan receptor GPR37L1 contributes to the sexual dimorphism of central cardiovascular control
Source: Biol Sex Differ. 2018 Apr 6;9:14. doi: 10.1186/s13293-018-0173-y (PMC5889568; doi:10.1186/s13293-018-0173-y)
Supplement: Supplementary file 1 — List of Supplemental Digital Content. (PDF 2673 kb) [file 13293_2018_173_MOESM1_ESM.pdf]

Online/Data Supplement

Orphan receptor GPR37L1 contributes to the sexual dimorphism of central cardiovascular control

James L.J. Coleman[a,b,c], Margaret A. Mouat[a,b,c], Jianxin Wu[c,d], Nikola Jancovski[e,f], Jaspreet K. Bassi[e], Andrea Y. Chan[c], David T. Humphreys[b,g], Nadine Mrad[a], Ze-Yan Yu[b,d], Tony Ngo[a,b,c], Siiri Iismaa[b,c], Cristobal G. dos Remedios[h], Michael P. Feneley[b,d], Andrew M. Allen[e,f], Robert M. Graham[b,c], Nicola J. Smith[a,b,c*].

Supplementary Figures and Tables

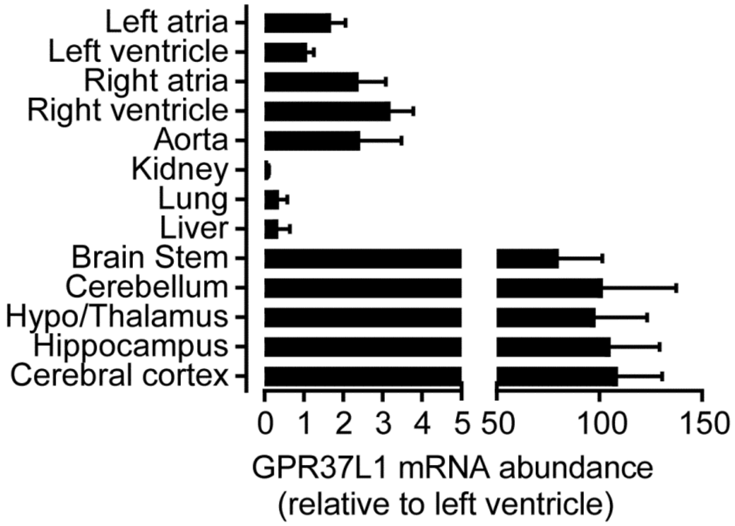

Supplementary Figure 1.

Supplementary Figure 1. GPR37L1 mRNA is abundant in the central nervous system. qPCR was performed on GPR37L1 in mouse tissues (as indicated) and expressed relative to mRNA abundance in the left ventricle. (10-12 week-old, male, n≥4).

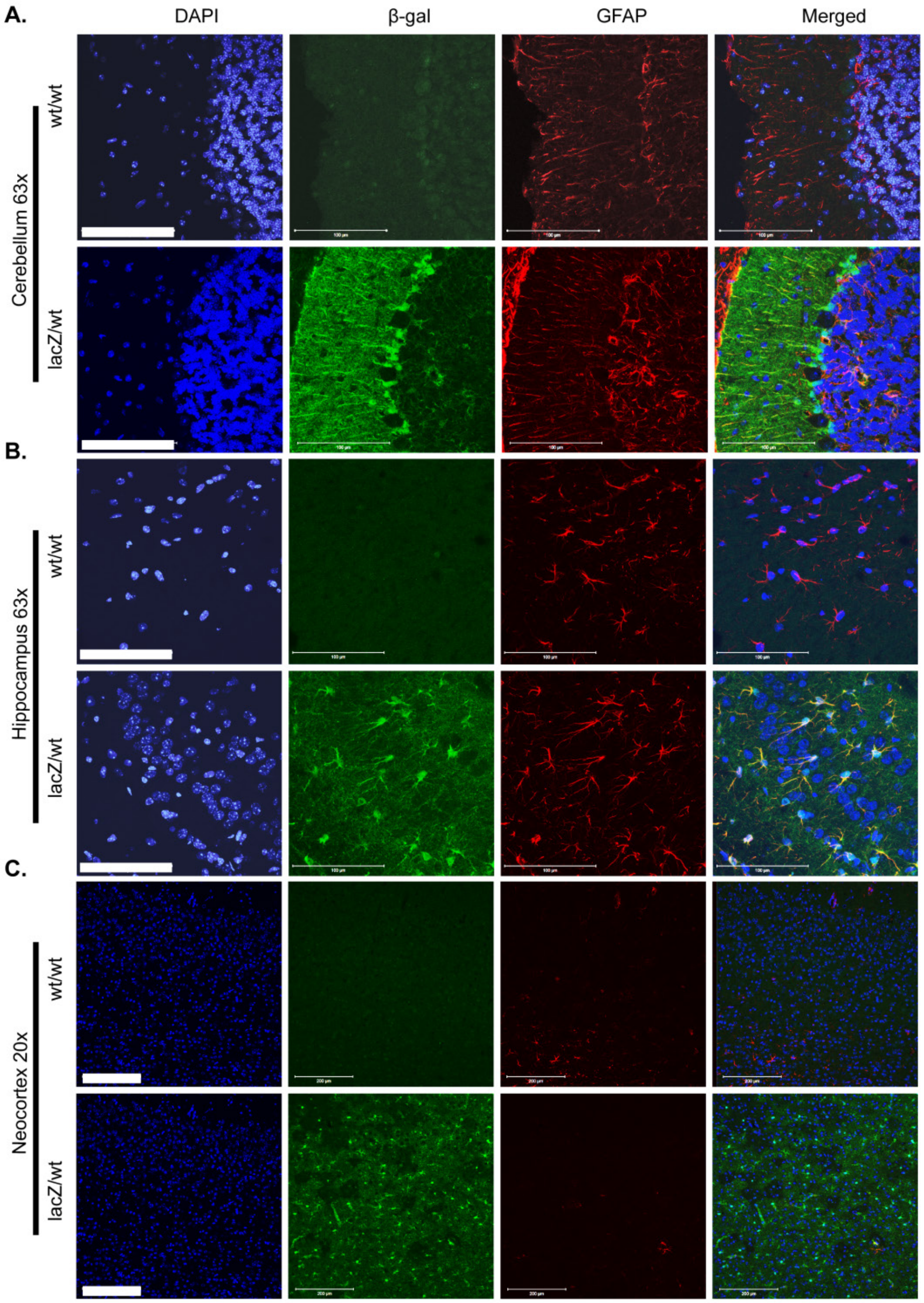

Supplementary Figure 2.

Supplementary Figure 2. Expression profile of GPR37L1 within the cerebellum, hippocampus and neocortex. **A-C.** Paraformaldehyde-fixed brain, heart and kidney tissue from GPR37L1$^{lacZ/wt}$ and GPR37L1$^{wt/wt}$ mice (mixed sex, ≥ 12 week-old) was embedded in

tissue-freezing medium, frozen and cut into 12 μm sections for immunohistochemistry and microscopy. In cerebellum (**A**), hippocampus (**B**) and neocortex (**C**): nuclei (blue, DAPI); GPR37L1 β-galactosidase reporter (green; β-galactosidase antibody); astrocytes and Bergmann glia [red, glial fibrillary acidic protein antibody (GFAP)]. Images are representative of n=3. Scale bar indicates: 100 μm in **A** and **B;** 200 μm in **C.**

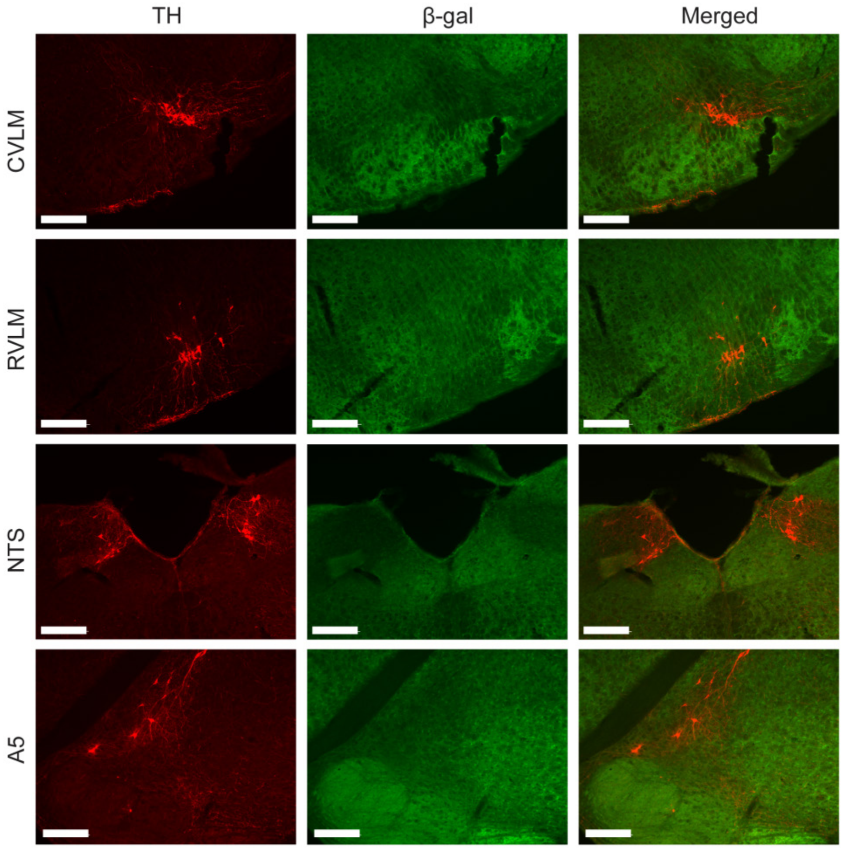

Supplementary Figure 3.

Supplementary Figure 3. Expression of GPR37L1 in central cardiovascular control regions: CVLM, RVLM, NTS and the A5 cluster. Paraformaldehyde-fixed brain tissue from a GPR37L1$^{lacZ/wt}$ mouse (male, ≥ 12 weeks of age, n=1) was embedded in tissue-freezing medium, frozen and cut into 20 μm sections for immunohistochemistry and microscopy. Catecholaminergic neurons were stained with antibody for tyrosine hydroxylase (TH; red) and GPR37L1 was stained with antibody for β-galactosidase (green). Scale bar indicates 500 μm. CVLM, caudo ventral lateral medulla; RVLM, rostral ventral lateral medulla; NTS, nucleus tractus solitarius.

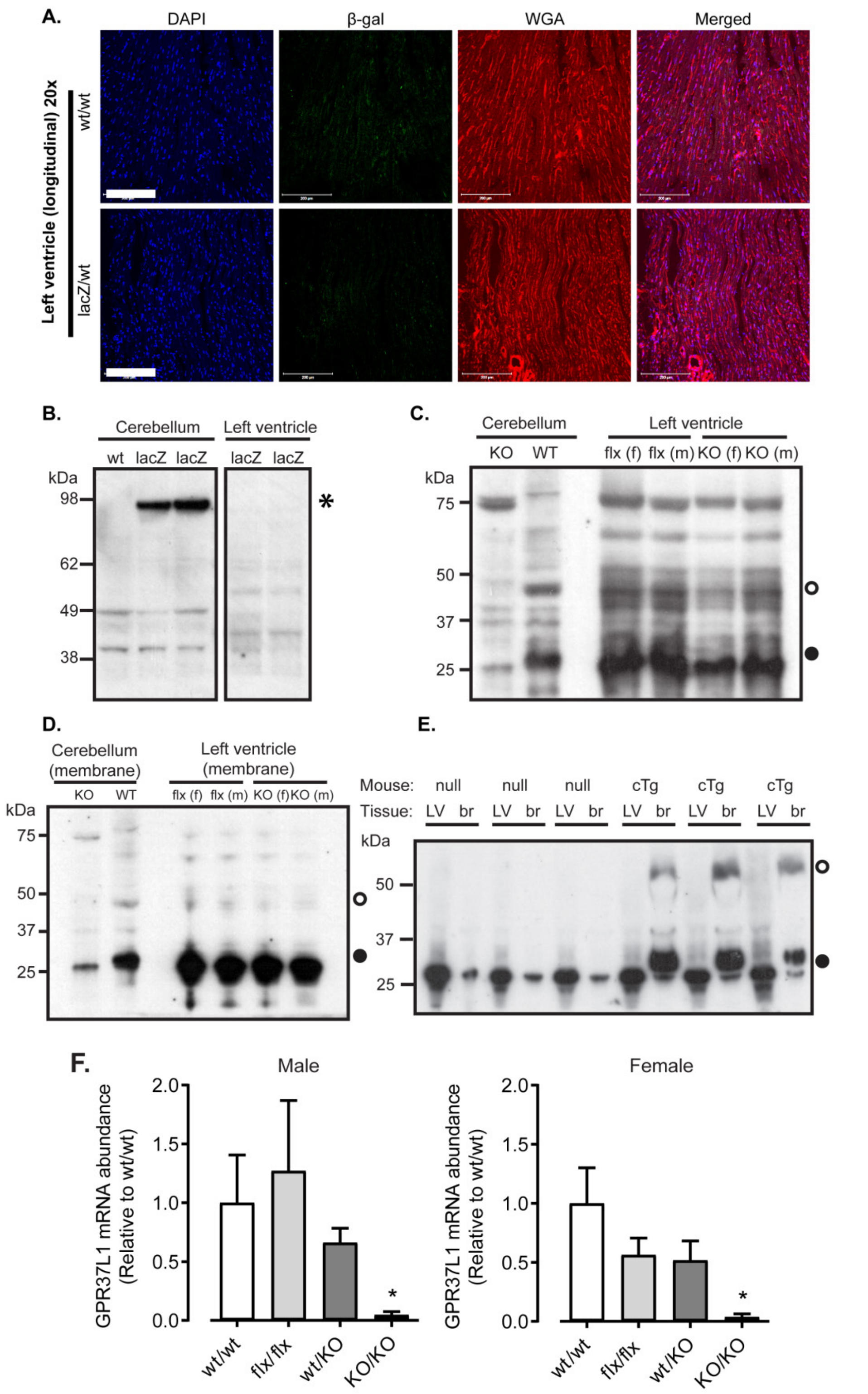

Supplementary Figure 4.

Supplementary Figure 4. Cardiac expression profile of GPR37L1. **A.** Heart tissue from $GPR37L1^{lacZ/wt}$ and $GPR37L1^{wt/wt}$ mice (mixed sex, ≥ 12 weeks of age) was embedded in tissue-freezing medium, frozen and cut into 12 μm sections for immunohistochemistry and microscopy. Nuclei were detected with DAPI stain (blue), β-galactosidase was detected by antibody (green) and cell membranes were detected with wheat germ agglutinin (WGA) stain (red). Images are representative of n=3. Scale bar indicates 200 μm. **B.** Immunoblot of cerebellar and left-ventricular (LV) homogenates from $GPR37L1^{lacZ/wt}$ and $GPR37L1^{wt/wt}$ mice (mixed-sex, ≥ 12 weeks of age). β-galactosidase was detected in $GPR37L1^{lacZ/wt}$ cerebellar lysates (asterisk, M_r of β-galactosidase, ~ 100 kD) but not in LV. Image shows n=2. **C.** Immunoblot of LV homogenates prepared from $GPR37L1^{flx/flx}$ and $GPR37L1^{KO/KO}$ mice (male and female as indicated, 10-12 weeks of age). Cerebellar lysates shown as antibody specificity controls. Image shows n=2. (open circle, M_r of full-length GPR37L1, ~50 kD; closed circle, M_r of cleaved GPR37L1, ~30 kD) **D.** Immunoblot of LV cell membranes prepared from $GPR37L1^{flx/flx}$ and $GPR37L1^{KO/KO}$ mice (male and female as indicated, 10-12 weeks of age). Cerebellar membrane preparations shown as antibody specificity controls. Image shows n=2. (open circle, M_r of full-length GPR37L1, ~50 kD; closed circle, M_r of cleaved GPR37L1, ~30 kD). **E.** Immunoblot of brain and LV homogenate prepared from $GPR37L1^{KO/KO}$ and GPR37L1-cTg [cardiomyocyte-specific GPR37L1-overexpressor, as reported previously[1]]. Mixed sex, 7-8 week-old. (open circle, M_r of full-length GPR37L1, ~50 kD; closed circle, M_r of cleaved GPR37L1, ~30 kD). **F.** qPCR was performed on LV from $GPR37L1^{wt/wt}$, $GPR37L1^{flx/flx}$, $GPR37L1^{wt/KO}$ and $GPR37L1^{KO/KO}$ mice, to confirm abrogation of cardiac GPR37L1 transcript in male and female $GPR37L1^{KO/KO}$ mice (10-12 weeks age, n=12, one-way ANOVA with Dunnett's multiple comparisons test,

*$p \leq 0.05$). Transcript abundance is expressed as a proportion of the GPR37L1$^{wt/wt}$ level within each sex.

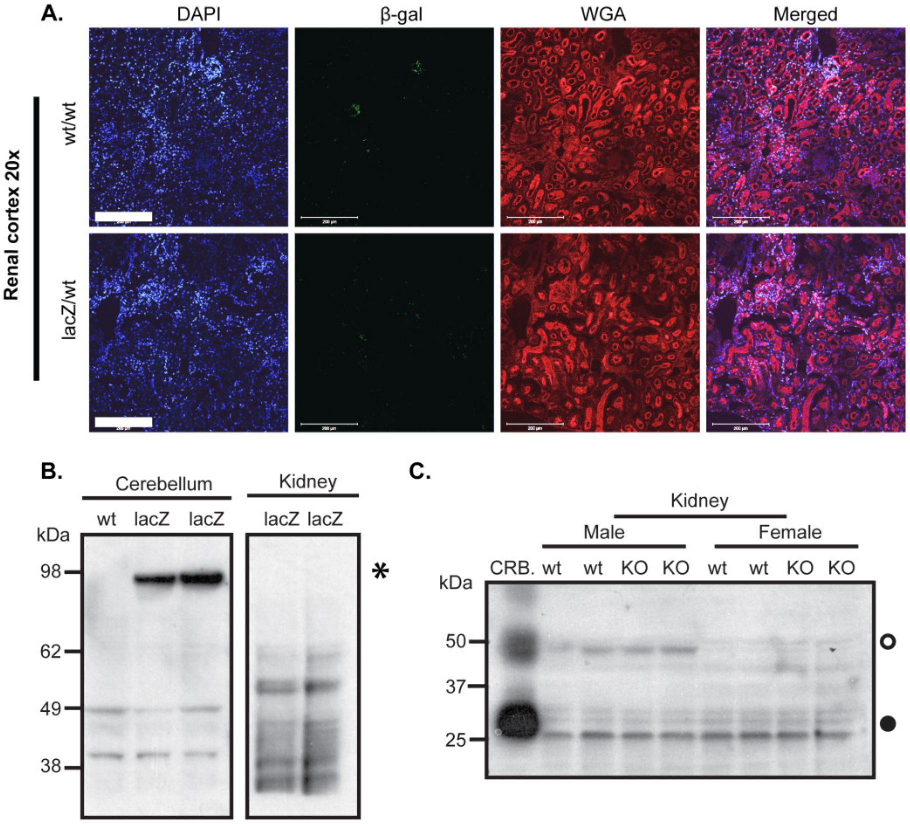

Supplementary Figure 5.

Supplementary Figure 5. Renal expression of GPR37L1. **A.** Kidney tissue from GPR37L1$^{lacZ/wt}$ and GPR37L1$^{wt/wt}$ mice (mixed-sex, ≥ 12 weeks of age) was embedded in tissue-freezing medium, frozen and cut into 12 µm sections for immunohistochemistry and microscopy. Nuclei were detected with DAPI stain (blue), β-galactosidase was detected by antibody (green) and cell membranes were detected with WGA stain (red). Images are representative of n=3. Scale bar indicates 200 µm. **B.** Immunoblot of cerebellar and kidney homogenates from GPR37L1$^{lacZ/wt}$ and GPR37L1$^{wt/wt}$ mice (mixed-sex, ≥ 12 weeks of age). β-galactosidase was detected in GPR37L1$^{lacZ/wt}$ cerebellar lysates (asterisk, M_r of β-

galactosidase, ~ 100 kD) but not in kidney. Immunoblot cropped from same exposure as that used in **Figure S4B,** and thus the cerebellar control tissue is duplicated. Image shows n=2. **C.** Immunoblot of kidney cell membranes prepared from GPR37L1$^{wt/wt}$ and GPR37L1$^{KO/KO}$ mice (male and female as indicated, 10-12 weeks of age). Cerebellar (CBR) membrane preparations shown as antibody specificity controls. Image shows n=2. (open circle, M_r of full-length GPR37L1, ~50 kD; closed circle, M_r of cleaved GPR37L1, ~30 kD).

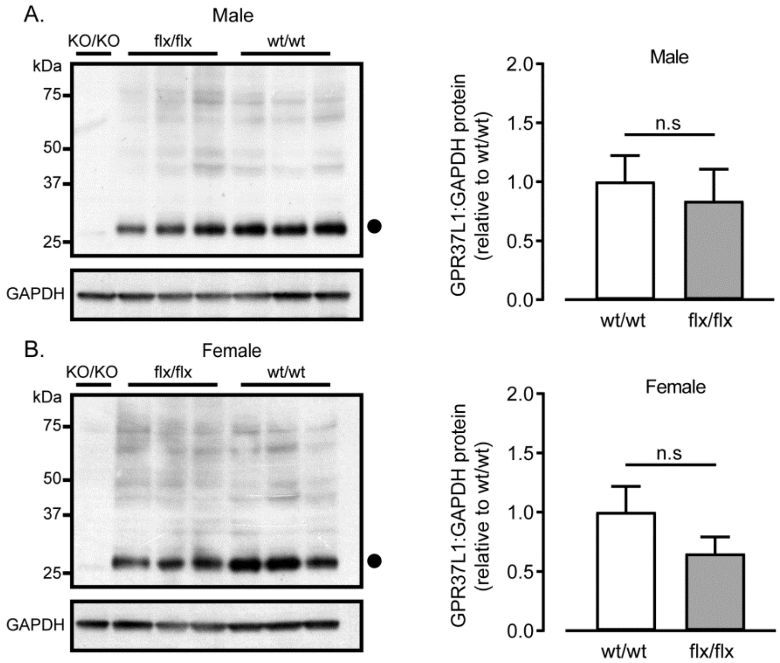

Supplementary Figure 6.

Supplementary Figure 6. Abundance of cerebellar GPR37L1 is unchanged in the GPR37L1$^{flx/flx}$ genotype. Immunoblot and densitometry (normalized to GAPDH) of GPR37L1 in cerebellar homogenate of GPR37L1$^{flx/flx}$ and GPR37L1$^{wt/wt}$ male (**A**) and female (**B**) mice (10-12 weeks age, n=3, two-tailed Student's t-test). Densitometry was performed on the predominating, cleaved GPR37L1 species (indicated by closed circle, M_r~30 kD).

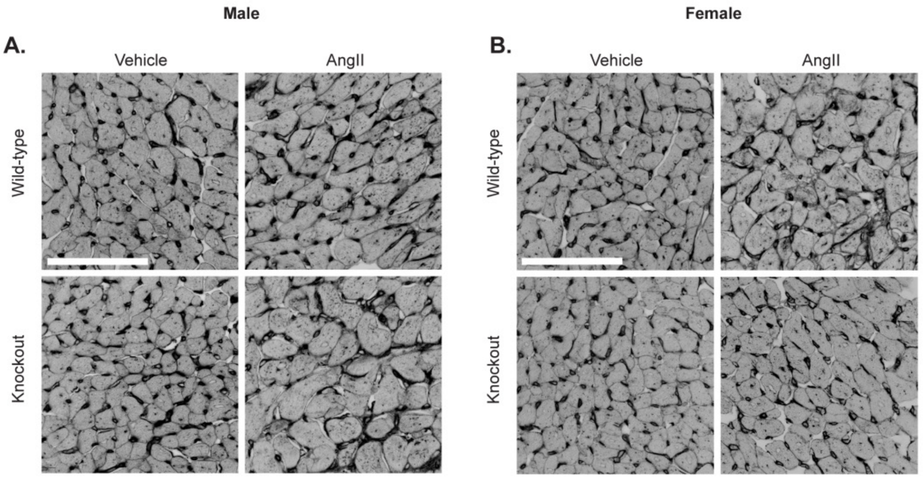

Supplementary Figure 7.

Supplementary Figure 7. Representative images of cardiomyocyte density in LV of AngII-treated mice. Paraformaldehyde-fixed LV tissue from male (**A.**) and female (**B.**) vehicle and AngII-treated GPR37L1$^{wt/wt}$ and GPR37L1$^{KO/KO}$ mice was cut into 12 μm sections and stained with WGA to highlight cell membranes. Images displayed in grey scale. Representative images of n≥6. Scale bar=100 μm. Quantification of cardiomyocyte density is shown in **Figure 3**.

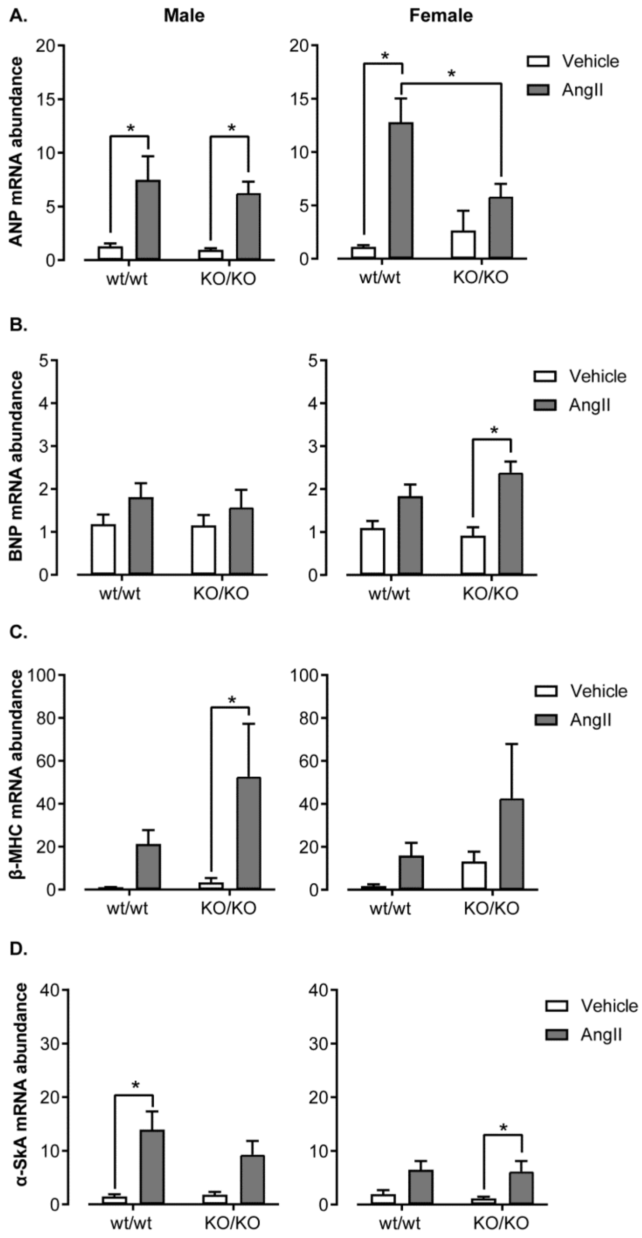

Supplementary Figure 8.

Supplementary Figure 8. Quantitative PCR analysis of cardiac fetal gene program mRNA in vehicle and AngII treated mice. Following 7 days of vehicle or AngII-infusion (2 mg/kg/day) LVs were harvested and cDNA was prepared for analysis of (**A**) ANP, (**B**) BNP, (**C**) β-MHC and (**D**) α-SkA. Transcript abundance normalized to β2M and expressed as

proportion of vehicle-infused GPR37L1$^{wt/wt}$ mice within each sex. n≥9. For **A-D**, two-way ANOVA results displayed in **Table S2**. *p≤0.05 according to Tukey's multiple comparison's test. ANP, atrial natriuretic peptide; BNP, brain natriuretic peptide; β-MHC, Beta myosin heavy chain; α-SkA, alpha skeletal actin; β2M, Beta-2 microglobulin.

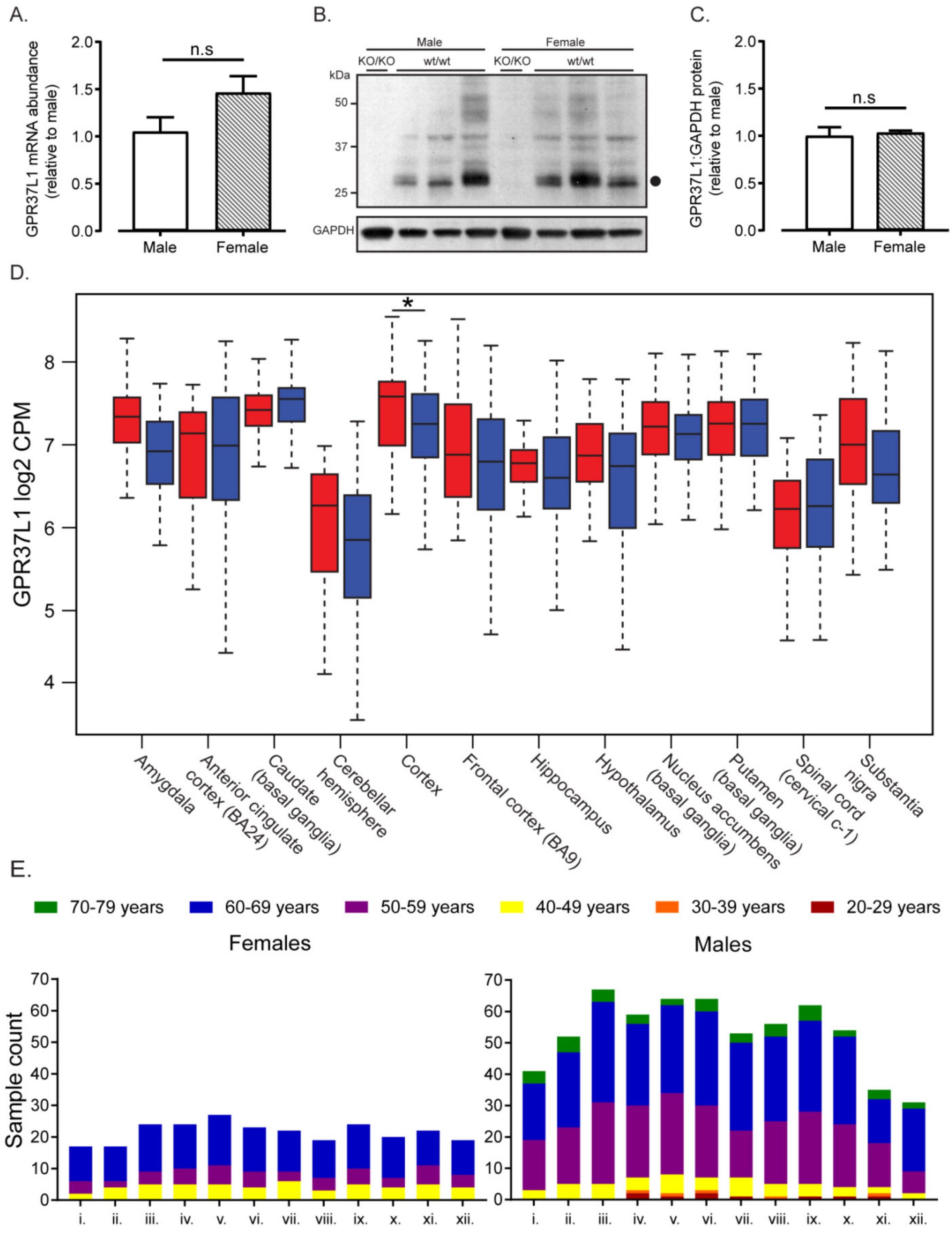

Supplementary Figure 9.

Supplementary Figure 9. Comparison of male and female GPR37L1 expression in mouse and human. **A.** qPCR was performed on cerebellum from male and female GPR37L1$^{wt/wt}$ mice (10-12 weeks age, n=6, two-tailed Student's t-test). **B.** GPR37L1 immunoblot and (**C**) densitometry of male and female GPR37L1$^{wt/wt}$ cerebellar homogenate (10-12 weeks age, n=3, two-tailed Student's t-test). Densitometry was performed on the predominating, cleaved GPR37L1 species (indicated by closed circle, M_r*~30 kD*). **D.** log2 GPR37L1 mRNA counts per million (CPM) in a heterogeneous cohort of human postmortem brain tissue (female, red, n=17-27; male, blue, n=31-68), as sequenced by the Genotype-Tissue Expression Consortium (GTEx)[2]. GTEx raw sequence counts were examined for differentially expressed genes using the edgeR statistical package[3], where samples were grouped by genders for each brain tissue sample. EdgeR statistical output for GPR37L1 was analyzed. * $p \leq 0.05$ between female and male samples within the same brain region. Expanded data shown in **Table S4** and age distribution of samples shown in panel **E.** (i. Amygdala, ii. Anterior cingulate cortex, iii. Caudate, iv. Cerebellar hemisphere, v. Cortex, vi. Frontal cortex, vii. Hippocampus, viii. Hypothalamus, ix. Nucleus accumbens, x. Putamen, xi. Spinal cord, xii. Substantia nigra).

Supplementary Table 1

Blood pressure by micromanometry in GPR37L1 knockout mice.

		Male			Female		
		**wt/wt**	**wt/KO**	**KO/KO**	**wt/wt**	**wt/KO**	**KO/KO**
**Aorta**	**HR** (bpm)	498.7±2.1	499±3.3	497.3±1.4	497.3±3.0	499.2±1.2	491.5±4.1
	**SP** (mmHg)	113.1±2.3	115.5±2.3	109.7±2.1	107.7±2.3	118±3.5*	119.3±2.7*
	**DP** (mmHg)	80.4±1.6	79.8±1.8	77.9±1.4	78.0±2.3	85.1±2.4	85.9±2.0*
	**MAP** (mmHg)	91.3±1.8	91.6±1.9	88.5±1.6	88.0±2.2	96.0±2.7	97.0±2.2*
	**PP** (mmHg)	32.7±1.0	35.7±1.2	31.8±1.1	29.7±1.1	32.9±1.2	33.4±1.1*
**LV**	**HR** (bpm)	500.4±1.0	493.4±2.3	500.6±1.1	501.7±1.9	496.7±2.2	489.3±4.6
	**SP** (mmHg)	111.3±2.2	113.1±2.0	113.2±3.0	107.7±2.4	111.1±2.8	116.1±2.5
	**EDP** (mmHg)	8.4±0.8	8.6±0.8	8.1±0.6	7.46±0.4	8.1±0.6	8.1±0.4
	**dP/dT max** (mmHg/s)	10957± 696.9	10574± 447.9	10577± 429.5	10406± 523.7	10265± 495.5	11262± 813.6
	**dP/dT min** (mmHg/s)	-9879± 501	-10731± 522.4	-10326± 566.5	-9088± 497.3	-9243± 473.4	-9575± 540.7

Underline and * indicates a significant ($p \leq 0.05$) difference as compared to $GPR37L1^{wt/wt}$ within each sex. One-way ANOVA with Dunnett's post-hoc test or Kruskal-Wallis with Dunn's multiple comparisons test where ANOVA assumptions were violated. HR, heart rate; SP, systolic pressure; DP, diastolic pressure; MAP, mean arterial pressure; PP, pulse pressure; EDP, end-diastolic pressure. n≥13 per genotype, per sex.

Supplementary Table 2. Expanded statistical analysis (two-way ANOVA) of **Figure S8** showing F statistic and P value of interaction, genotype and AngII effects on ANP, BNP, β-MHC and α-SkA mRNA abundance as determined by qPCR. Underline highlights p ≤0.05.

		**Males**		**Females**	
**Parameter**	**Effect**	**F (DFn, DFd)**	**P value**	**F (DFn, DFd)**	**P value**
**ANP**	**Interaction**	F (1, 37) = 0.12	P=0.732	F (1, 35) = 7.74	<u>P<0.001</u>
	**Genotype**	F (1, 37) = 0.35	P=0.560	F (1, 35) = 3.14	P=0.085
	**AngII**	F (1, 37) = 19.47	<u>P<0.001</u>	F (1, 35) = 23.53	<u>P<0.001</u>
**BNP**	**Interaction**	F (1, 37) = 0.11	P=0.738	F (1, 35) = 2.65	P=0.113
	**Genotype**	F (1, 37) = 0.19	P=0.663	F (1, 35) = 0.69	P=0.414
	**AngII**	F (1, 37) = 2.87	P=0.099	F (1, 35) = 24.24	<u>P<0.001</u>
**β-MHC**	**Interaction**	F (1, 37) = 1.35	P=0.258	F (1, 35) = 0.32	P=0.577
	**Genotype**	F (1, 37) = 1.80	P=0.188	F (1, 35) = 1.99	P=0.167
	**AngII**	F (1, 37) = 7.69	<u>P=0.009</u>	F (1, 35) = 2.60	P=0.116
**α-SkA**	**Interaction**	F (1, 37) = 1.20	P=0.274	F (1, 35) = 0.03	P=0.860
	**Genotype**	F (1, 37) = 0.92	P=0.344	F (1, 35) = 0.18	P=0.677
	**AngII**	F (1, 37) = 19.59	<u>P<0.001</u>	F (1, 35) = 13.53	<u>P<0.001</u>

ANP, atrial natriuretic peptide; BNP, brain natriuretic peptide; β-MHC, Beta myosin heavy chain; α-SkA, alpha skeletal actin.

Supplementary Table 3. Expanded statistical analysis (two-way ANOVA) of **Tables 1&2**, showing F statistic and P value of interaction, genotype and AngII effects on hemodynamic and histological parameters. Underline highlights p ≤0.05.

		**Males**		**Females**	
**Parameter**	**Effect**	**F (DFn, DFd)**	**P value**	**F (DFn, DFd)**	**P value**
**Aortic HR**	**Interaction**	F (1, 32) = 0.08	P=0.782	F (1, 33) = 0.04	P=0.852
	**Genotype**	F (1, 32) = 0.06	P=0.814	F (1, 33) = 2.38	P=0.139
	**AngII**	F (1, 32) = 1.08	P=0.307	F (1, 33) = 0.10	P=0.760
**Aortic SP**	**Interaction**	F (1, 32) = 0.05	P=0.828	F (1, 33) = 2.81	P=0.103
	**Genotype**	F (1, 32) = 1.65	P=0.208	F (1, 33) = 0.42	P=0.522
	**AngII**	F (1, 32) = 4.27	<u>P=0.047</u>	F (1, 33) = 17.24	<u>P<0.001</u>
**Aortic DP**	**Interaction**	F (1, 32) = 0.07	P=0.791	F (1, 33) = 0.15	P=0.670
	**Genotype**	F (1, 32) = 0.82	P=0.373	F (1, 33) = 2.62	P=0.115
	**AngII**	F (1, 32) = 0.43	P=0.518	F (1, 33) = 3.45	P=0.072
**Aortic MAP**	**Interaction**	F (1, 32) = 0.07	P=0.801	F (1, 33) = 1.08	P=0.307
	**Genotype**	F (1, 32) = 1.15	P=0.292	F (1, 33) = 1.46	P=0.235
	**AngII**	F (1, 32) = 1.47	P=0.235	F (1, 33) = 9.39	<u>P<0.001</u>
**Aortic PP**	**Interaction**	F (1, 32) < 0.01	P=0.994	F (1, 33) = 5.70	<u>P=0.023</u>
	**Genotype**	F (1, 32) = 1.53	P=0.225	F (1, 33) = 0.19	P=0.672
	**AngII**	F (1, 32) = 12.45	<u>P=0.001</u>	F (1, 33) = 25.99	<u>P<0.001</u>
**LV HR**	**Interaction**	F (1, 31) = 0.80	P=0.378	F (1, 31) = 0.12	P=0.733
	**Genotype**	F (1, 31) = 0.44	P=0.511	F (1, 31) = 2.58	P=0.118

	**AngII**	F (1, 31) = 0.81	P=0.375	F (1, 31) = 0.18	P=0.677
**LV SP**	**Interaction**	F (1, 31) = 0.87	P=0.359	F (1, 31) = 1.92	P=0.176
	**Genotype**	F (1, 31) = 3.29	P=0.080	F (1, 31) = 0.38	P=0.540
	**AngII**	F (1, 31) = 3.71	P=0.064	F (1, 31) = 31.92	P<0.001
**LV EDP**	**Interaction**	F (1, 31) = 1.50	P=0.231	F (1, 31) = 2.64	P=0.114
	**Genotype**	F (1, 31) = 0.06	P=0.811	F (1, 31) = 0.10	P=0.754
	**AngII**	F (1, 31) = 1.09	P=0.305	F (1, 31) = 5.47	P=0.026
**LV dP/dT max**	**Interaction**	F (1, 31) = 2.28	P=0.142	F (1, 31) < 0.01	P=0.945
	**Genotype**	F (1, 31) = 12.03	P=0.002	F (1, 31) = 5.71	P=0.023
	**AngII**	F (1, 31) = 5.95	P=0.021	F (1, 31) = 3.39	P=0.075
**LV dP/dT min**	**Interaction**	F (1, 31) = 0.02	P=0.891	F (1, 31) = 0.04	P=0.851
	**Genotype**	F (1, 31) = 2.36	P=0.135	F (1, 31) = 0.36	P=0.553
	**AngII**	F (1, 31) = 1.71	P=0.200	F (1, 31) = 0.34	P=0.563
**HW:TL**	**Interaction**	F (1, 86) = 5.25	P=0.024	F (1, 69) = 0.35	P=0.557
	**Genotype**	F (1, 86) = 13.44	P<0.001	F (1, 69) = 2.13	P=0.149
	**AngII**	F (1, 86) = 40.15	P<0.001	F (1, 69) = 86	P<0.001
**LVW:TL**	**Interaction**	F (1, 86) = 6.27	P=0.014	F (1, 69) = 0.33	P=0.569
	**Genotype**	F (1, 86) = 19.17	P<0.001	F (1, 69) = 5.59	P=0.021
	**AngII**	F (1, 86) = 67.31	P<0.001	F (1, 69) = 129.7	P<0.001
**RVW:TL**	**Interaction**	F (1, 86) = 0.49	P=0.485	F (1, 69) = 0.48	P=0.489
	**Genotype**	F (1, 86) = 1.86	P=0.176	F (1, 69) = 0.45	P=0.507
	**AngII**	F (1, 86) = 2.22	P=0.140	F (1, 69) = 0.51	P=0.477

**Lung:TL**	**Interaction**	F (1, 81) = 3.79	P=0.055	F (1, 69) = 0.91	P=0.344
	**Genotype**	F (1, 81) = 0.93	P=0.337	F (1, 69) = 0.13	P=0.717
	**AngII**	F (1, 81) = 2.36	P=0.128	F (1, 69) < 0.01	P=0.986
**CM density**	**Interaction**	F (1, 22) = 3.06	P=0.094	F (1, 22) = 0.16	P=0.694
	**Genotype**	F (1, 22) = 0.53	P=0.473	F (1, 22) = 2.16	P=0.156
	**AngII**	F (1, 22) = 12.02	P=0.002	F (1, 22) = 25.15	P<0.001
**Fibrosis**	**Interaction**	F (1, 21) = 3.77	P=0.066	F (1, 24) = 10.3	P=0.009
	**Genotype**	F (1, 21) = 0.71	P=0.408	F (1, 24) = 2.02	P=0.168
	**AngII**	F (1, 21) = 17.64	P<0.001	F (1, 24) = 0.53	P=0.474

HR, heart rate; SP, systolic pressure; DP, diastolic pressure; MAP, mean arterial pressure; PP, pulse pressure; EDP, end-diastolic pressure; HW, heart weight; LVW, left ventricle weight; TL, tibia length; LV, left ventricle; RVW, right ventricle weight; CM, cardiomyocyte.

Supplementary Table 4. Expanded human postmortem brain RNA sequencing data from **Figure S9.** Underline highlights p ≤0.05.

	Average GPR37L1 log2 CPM	GPR37L1 F:M log2 FC	P value	FDR
**Amygdala**	7.1397	0.0984	0.5442	0.8607
**Anterior cingulate cortex (BA24)**	7.1970	0.0244	0.9004	1.0000
**Caudate (basal ganglia)**	7.5656	-0.1556	0.2872	0.9995
**Cerebellar hemisphere**	6.0750	0.2972	0.1078	0.3994
**Cortex**	7.3813	0.3512	<u>0.0174</u>	0.4502
**Frontal cortex (BA9)**	7.1313	0.1566	0.4767	0.9998
**Hippocampus**	6.7679	-0.0209	0.9016	0.9854
**Hypothalamus**	6.7878	0.3117	0.0644	1.0000
**Nucleus accumbens (basal ganglia)**	7.2684	0.0417	0.7756	0.9534
**Putamen (basal ganglia)**	7.3438	-0.2939	0.0703	0.7201
**Spinal cord (cervical c-1)**	6.3156	-0.1612	0.3068	1.0000
**Substantia nigra**	6.9968	0.2161	0.2657	0.9957

CPM, counts per million; FC, fold change; FDR, false discovery rate; F, female; M, male.

1. Min KD, Asakura M, Liao Y, Nakamaru K, Okazaki H, Takahashi T, et al. Identification of genes related to heart failure using global gene expression profiling of human failing myocardium. Biochemical and biophysical research communications 2010; 393 (1):55-60.

2. Consortium GT. Human genomics. The Genotype-Tissue Expression (GTEx) pilot analysis: multitissue gene regulation in humans. Science 2015; 348 (6235):648-660.

3. Robinson MD, McCarthy DJ, Smyth GK. edgeR: a Bioconductor package for differential expression analysis of digital gene expression data. Bioinformatics 2010; 26 (1):139-140.
